# A novel de novo *CLTC* variant altering RNA splicing causes fetal developmental abnormalities

**DOI:** 10.1186/s12920-023-01778-3

**Published:** 2023-12-18

**Authors:** Chen Cheng, Fan Yang, Sheng Zhao, Xinlin Chen

**Affiliations:** Ultrasound Diagnosis Department, Maternal and Child Health Hospital of Hubei Province, Wuhan, 430070 China

**Keywords:** *CLTC*, Fetus, Prenatal diagnosis, Choroid plexus cysts, RNA splicing

## Abstract

**Background:**

About 31 individuals with *CLTC* variants have been reported worldwide, and all reported individuals have motor and mental retardation. *CLTC* is known to lead to intellectual developmental disorder, autosomal dominant 56. Few studies are focusing on the prenatal stage of the disease.

**Method:**

An ultrasound examination was performed to obtain the prenatal phenotype. Whole-exome sequencing was used to find the pathogenic variant. Multiple computational tools predicted the conservation and deleteriousness. Minigene assay and western blot were utilized to investigate the effect on splicing of mRNA and protein expression.

**Result:**

Here we found a novel de novo variant of *CLTC* in a fetus. The fetus manifested bilateral choroid plexus cysts of the brain, hyperechogenic kidneys, and ventricular septal defect. A heterozygous variant c.3249 + 1G > C was identified in the fetus. This position was conserved and the variant was predicted to be deleterious. Minigene assay revealed the presence of a truncating transcript with the retention of intron 20. Western blot result showed the c.3249 + 1G > C variant elicited degradation of the protein.

**Conclusion:**

To the best of our knowledge, our study identified a novel de novo variant of *CLTC* and provided the earliest clinical characteristic of the *CLTC* variant at the prenatal stage. The functional experiment suggested the variant caused the altering of the RNA splicing and the protein expression. We extended the mutational spectrum of *CLTC* and provided guidance on genetic counseling.

**Supplementary Information:**

The online version contains supplementary material available at 10.1186/s12920-023-01778-3.

## Introduction

Clathrin heavy chain (CLTC) is a triskelion scaffolding protein of clathrin-coated vesicles, conducting trafficking and endocytosis function [1]. Clathrin is evolutionarily conserved and highly expressed in multiple tissues participating in a diverse range of cellular functions [2]. De novo *CLTC* variants are associated with a variable phenotype from mild to severe intellectual disability, microcephaly, hypoplasia of the corpus callosum, and epilepsy [3–5].

About 31 individuals with *CLTC* pathogenic variants have been reported worldwide, and all reported individuals have motor and mental retardation [6]. The present reported patients are limited and the relationship between genotype and phenotype remains to be revealed. The manifestations of *CLTC* variants were complex and involved multiple systems. The reported uncommon complications included epilepsy, ophthalmopathy, gastrointestinal abnormalities, cerebral hemorrhage, neurocytoma, and Parkinson’s disease. However, few studies focused on the manifestation of the prenatal stage. The fetus with *CLTC* c.2737_2738dupGA was large for gestational age at 35 weeks [3]. The individual harboring c.3662_3664del of *CLTC* showed fetal growth restriction at 18 weeks of gestation initially [6]. *CLTC* c.3662_3664del and c.2430 + 1G > T was first reported to cause brain defect (cystic lesions along the lateral ventricles of the brain) and kidney phenotypes (high-echogenic kidneys, agenesis of the left kidney and right vesicoureteral reflux) in 2022 [6].

Here we found a novel de novo variant of *CLTC* in a fetus, continuous clinical ultrasound prenatal manifestations were recorded and a functional experiment of the variant was studied. We also compared the fetus with the previously reported one.

## Materials and methods

### Ultrasound examination

The patient was diagnosed by a qualified sonographer and confirmed by a senior prenatal sonographer. Samsung WS80A and Mindray Resona R9 machines were used to perform the examination and CA1-7 A, and SC6-1U transducers were utilized.

### Clinical trio-whole exome sequencing (WES)

Clinical Trio-WES was performed in the BGI Company, Wuhan, China. Genomic DNA was obtained from the patient’s blood. Firstly, the DNA was broken and the library was prepared, then the DNA in the exons of the target gene and the adjacent shear region was captured and enriched by the Roche KAPA Hyper Exome chip, and finally, the MGISEQ-2000 sequencing platform was used for variant detection. The average sequencing depth of the target region was ≥ 180X, among which the average depth of the target region > 20X loci accounted for > 95%. The proportion of loci in the target region > 20X was > 95%. Sequenced fragments were aligned to the UCSC hg19 human reference genome by BWA to remove duplicates. The correction was performed using GATK SNV, INDEL, and genotype detection. ExomeDepth was used for copy number variation detection at the exon level. The classification of variant pathogenicity was based on the American College of Medical Genetics and Genomics (ACMG) and American Molecular Pathology Society (AMP) guidelines for the interpretation of sequence variants, concerning the ClinGen Sequence Variation Interpretation Working Group and the British Clinical Genomic Sciences Society (ACGS) for a refined interpretation of the guidelines [7, 8].

### Bioinformatic prediction

Multiple bioinformatic prediction tools were used to evaluate the variant including Mutation Taster [9]. The Single-Nucleotide Polymorphism database (dbSNP, https://www.ncbi.nlm.nih.gov/snp/), 1000 Genomes Project data (https://www.internationalgenome.org/), the Genome Aggregation Database (gnomAD, http://gnomad.broadinstitute.org) were used to find the variant frequency in populations. PhyloPvertebrates (http://compgen.cshl.edu/phast/) was used to predict the conservation of the variant. SpliceAI (https://spliceailookup.broadinstitute.org/) was used to predict the effect of splice variants.

### Variant validation by Sanger sequencing

The *CLTC* c.3249 + 1G > C variant was validated by Sanger sequencing. Polymerase chain reaction (PCR) amplification was conducted using the designed upstream primer “TGTCGGATAAACTTAAAATATTGCAT” and the downstream primer “CAACTGGGCTTTTGCAAGTT”.

### Minigene assay to explore alternative splicing

Genomic segments from the exon 19 to exon 21 of *CLTC* including wild-type and mutant (NM_004859.3:c.3249 + 1G > C) were constructed into the minigene vector pMini-CopGFP (Hitrobio. tech, China) using the Seamless Cloning Kit respectively. The upstream primer and downstream primer for the wild-type and mutant vector were as follows: CLTC-F, AAGCTTGGTACCGAGCTCGGATCCGTTGTACAAACAGCTTTGTCTGAGACTC; CLTC-R, TTAAACGGGCCCTCTAGACTCGAGCACTAGTATTGGCAGCCTGAACAACTTC; CLTC-MUT-F, CTTCAGCAGTTCAGcTAAATCTTCAGATTACCTAAGTTGAATTACTAA, CLTC-MUT-R, TAgCTGAACTGCTGAAGTATTGACATCAAATT. The *CLTC* wild-type minigene plasmid and *CLTC* mutant minigene plasmid were constructed and transfected with 293T cells respectively. cDNA was extracted from RNA reverse transcription and PCR amplified by analyzing PCR fragment size and PCR sequencing results.

### Western blot

The Wild (WT) and Mutant (MT) *CLTC* sequence of exon 19 to exon 23 (E19-E23) and exon 19 to exon 23 (E19-E21) with an N-terminal eGFP tag was cloned to the expression vectors. The plasmids were transfected to the HEK293t cells. The primary antibodies GFP Monoclonal antibody (66002-1-Ig, Proteintech Group) and GAPDH Monoclonal antibody (60004-1-Ig, Proteintech Group) were used. The second antibody used in the study was HRP-conjugated Affinipure Goat Anti-Mouse IgG (H + L) (SA00001-1, Proteintech Group).

## Results

### Clinical ultrasound findings of a fetus with developmental abnormalities

**Fig. 1:**
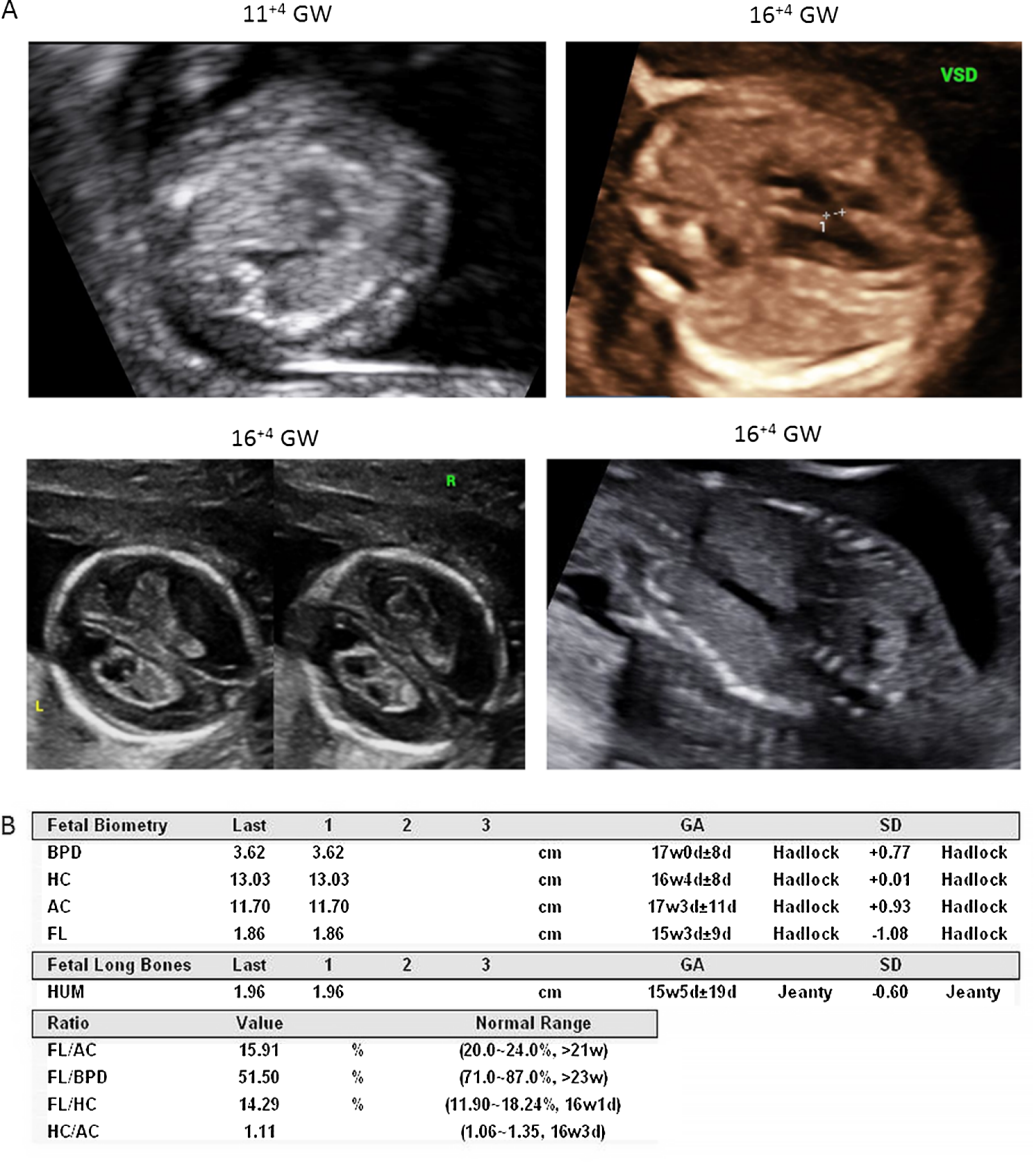
Fetal abnormalities found by sonographic scanning. **A** Sonographic scanning detected ventricular septal defect at 11 + 4 GW and VSD of 0.02 cm, bilateral choroid plexus cysts of the brain and hyperechogenic kidneys at 16 + 4 GW. **B** The fetal biometry including BPD, HC, AC, FL and HL, the ratio of FL/AC, FL/BPD, FL/HC, HC/AC were all in the normal range. BPD: biparietal diameter; HC: head circumference; AC: abdominal circumference; FL: femur length; HL: humerus length

The pregnant woman was referred to our hospital for early pregnancy ultrasound scanning. The fetus was suspected of a cardiac ventricular septal defect (VSD) of 0.02 cm at 11^+ 4^ gestational week (Fig. 1A). During the next scanning, bilateral choroid plexus cysts of the brain, hyperechogenic kidneys, and VSD appeared at 16^+ 4^ gestational week (Fig. 1A). The other fetal biometry including biparietal diameter (BPD), head circumference (HC), abdominal circumference (AC), femur length (FL), humerus length (HL), the ratio of FL/AC, FL/BPD, FL/HC, HC/AC were all in the normal range (Fig. 1B). The parents were healthy and nonconsanguineous with no genetic disease. The paternity was confirmed.

### **Identification of a de novo variant c.3249 + 1G > C of*CLTC*in the abnormal fetus of a trio-family**

**Fig. 2:**
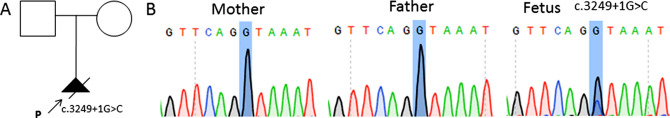
*CLTC* variant c.3249 + 1G > C associated with the fetal manifestation detected in the family. **A** Pedigree of the family. **B** Sanger sequencing spectra showing a de novo heterozygous variant c.3249 + 1 G > C in the fetus while the mother and father carrying the wild type

After the ultrasound diagnosis of 16 + 4 GW, the parents requested termination of the pregnancy. The postnatal WES test was conducted. Then the pregnancy was terminated and the umbilical cord blood was collected for the genetic test. The genomic DNA was extracted from the peripheral blood of the parents. The clinical trio-Whole Exome Sequencing was conducted in the family. A pathogenic variant was detected in the *CLTC* associated with autosomal dominant mental disorder type 56, which was partially related to the phenotype of the fetus. The variant was de novo and located at c.3249 + 1G > C (NM_004859.3) (Fig. 2A). This variant had not been reported previously. No other variants associated with clinical phenotypes were detected. Neither mitochondrial gene variants nor suspected pathogenic variations associated with clinical phenotypes were detected. Sanger sequencing was performed to validate the c.3249 + 1G > C variant (Fig. 2B). This variant was classified as “pathogenic” with the evidence of PVS1 + PS2_Supporting + PM2 from the ACMG criteria.

### **Computational predictions on the functional effects of*CLTC*c.3249 + 1G > C variation**

MutationTaster software predicted that c.3249 + 1G > C was disease-causing. No frequency was found in the Single-Nucleotide Polymorphism database (dbSNP), 1000 Genomes Project data, the Genome Aggregation Database (gnomAD). The computational tools (PhyloPvertebrates) predicted that c.3249 + 1G position was conserved (Table 1). Splicing prediction tools (SpliceAI) showed the c.3249 + 1G > C was deleterious and might cause abnormal splicing (Table 2).

**Table 1:** Conservation prediction of the variant by PhyloPvertebrates

HGVS-c	PhyloP vertebrates
c.3249 + 1G > C	6.199; Conserved

**Table 2:** Functional prediction of the variant by SpliceAI.

HGVS-c	SpliceAI	SpliceAI Pred	SpliceAI Interpretation
c.3249 + 1G > C	SpliceAI = C|CLTC|0.00|0.00|0.41|1.00|12|-47|20|-1	D	17:57758860 (= 57,758,840 + 20) donor gain0.41; 17:57758839 (= 57758840-1) donor loss 1.0

### **Functional analysis of the *CLTC *c.3249 + 1G > C variant using minigene assay**

**Fig. 3:**
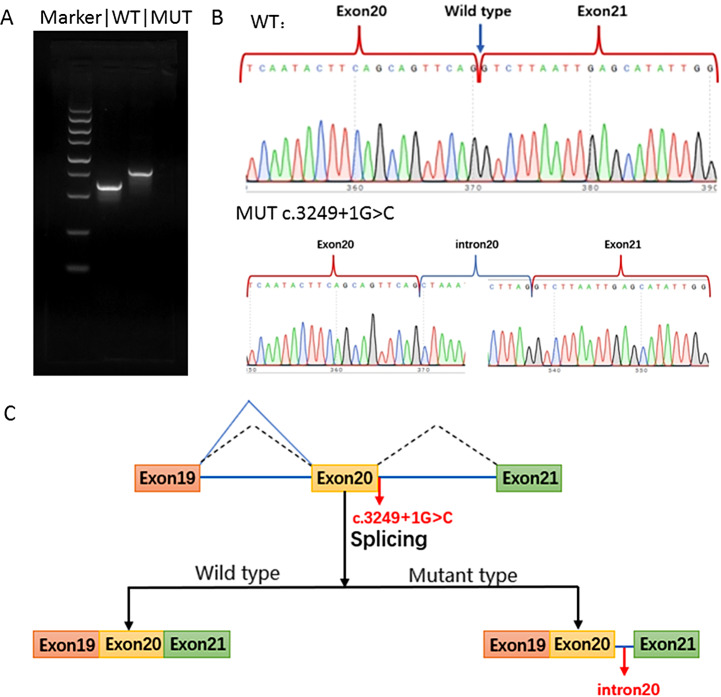
Minigene assay revealed the *CLTC* variant c.3249 + 1G > C altered the RNA splicing. **A** Agarose gel electrophoresis result showing that the mRNA of the MT was longer than the WT. **B** Sanger sequencing of the RT-PCR product showed the WT was 594 bp and MT was 762 bp with the retention of intron 20. **C** Schematic diagram showing the aberrant splicing

To access the splicing impact of the *CLTC* c.3249 + 1G > C variant, we used the minigene assay to functionally test the splicing effect. The variant at the exon-intron junction caused the presence of a transcript with the retention of intron 20. The agarose gel-electrophoresis result was shown in Fig. 3A. The predicted PCR amplification of the WT sequence length was 594 bp, while the MUT sequence length was 762 bp, with an extra 168 bp (intron 20). Sanger sequencing of the RT-PCR products validated that MUT caused mRNA retention of intron 20 (Fig. 3B). The schematic diagram illustrated aberrant splicing, as shown in Fig. 3C. The mutant mRNA product was represented as NM_004859.3: c.3249 + 1_3249 + 168ins. The predicted protein shifts code to form a truncated protein, and the protein was represented as p.Val1084LeufsTer7.

### Mutant CLTC caused significantly degradation of the protein

**Fig. 4:**
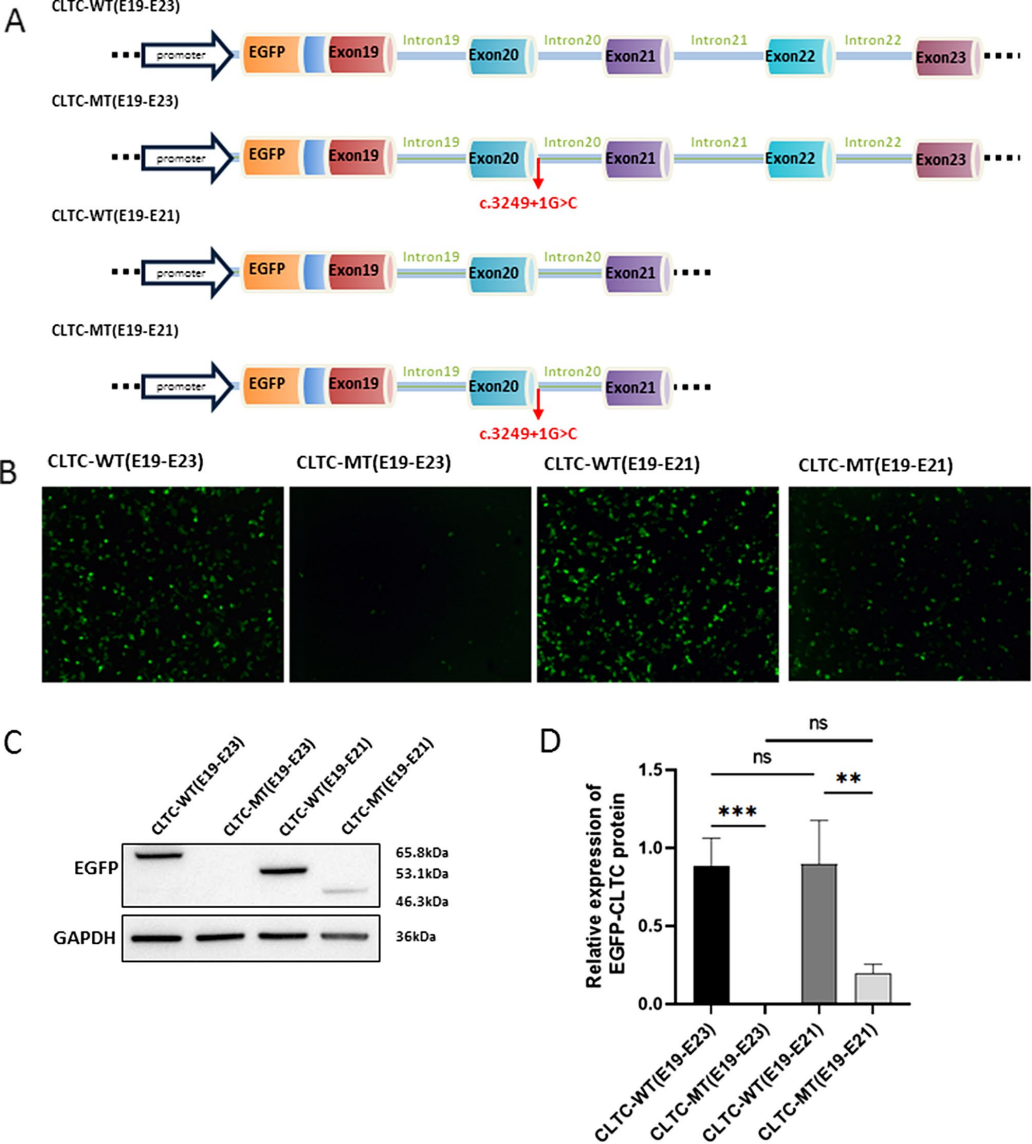
The MT of CLTC decreased the protein expression. **A** Schematic diagram of the fabricated plasmid from CLTC-WT and CLTC-MT with exon19 to exon23 (E19-E23) and exon19 to exon21 (E19-E21). **B-D** After transfection of the CLTC-coEGFP expression vector to the HEK293T, the MT (both exon 19-exon 21 and exon 19-23) was observed much lower than the WT. The predicted WT (E19-E23) was 65.8 kDa and the MT (p.Val1084LeufsTer7) was 46.3kDa. The predicted WT (E19-E21) was 53.1 kDa and the MT was 46.3 kDa

The Wild (WT) and Mutant (MT) *CLTC* sequence of exon 19 to exon 21 (E19-E21) and exon 19 to exon 23 (E21-E23) with an N-terminal GFP tag was cloned to the expression vectors (Fig. 4A). We investigated the effect of the c.3249 + 1G > C variant on the protein expression. Through the transfection and western blot experiment, we demonstrated the protein level of the abnormal transcript was much lower than the normal one (Fig. 4B-D). Thus, the nonsense-mediated decay of the truncated transcript was likely induced. To verify our hypothesis, we knocked down the UPF1 protein which was essential to the NMD process, however, the MT CLTC was not rescued (Data not shown).

## Discussion

In our study, the affected fetus harbored a novel de novo variant in *CLTC* which we demonstrated the pathogenicity of the variant. Till now few studies have focused on the manifestation of individuals with CLTC variants. Even less was reported at the prenatal stage. Multiple computational tools predicted the variant to be deleterious to cause mRNA splicing abnormality and functional experiments suggested that the variant introduced a premature termination codon. The c.3249 + 1G > C *CLTC* variant caused CLTC protein expression significantly degraded. The CLTC expression played an important role in multiple tissues. The CLTC protein structure is made up of 6 parts, including the Terminal domain, Ankle domain, Distal leg, Knee, Proximal leg, and Trimerization domain residues. The novel de novo heterozygous variant c.3249 + 1G > C caused the truncation of CLTC protein lacking Knee residues, Proximal leg residues, and Trimerization domain residues. Guided by the results of the western blot analysis, it was observed that the expression of the MT protein, spanning residues E19-E21, was notably reduced when compared to the wild-type protein (E19-E21). Additionally, the expression of the MT protein, spanning residues E19-E23, was also significantly decreased in comparison to the wild-type protein (E19-E23). Nevertheless, despite knocking down the UPF1 protein, the MT CLTC was not rescued. Further investigation into the underlying mechanism is needed. The c.3249 + 1G > C *CLTC* variant resulted in the LOF of the gene product. The clinical phenotype of the fetus was probably associated with the haploinsufficiency of full-length CLTC.

To the best of our knowledge, our study presented the earliest initial manifestations among individuals with *CLTC* variants. The fetus was observed by continuous ultrasound scanning. The initial cardiac VSD occurred in 11 GW, and subsequent bilateral choroid plexus cysts of the brain and hyperechogenic kidneys appeared in 16 GW. We compared the manifestations with the occurrence of gestational weeks between the fetus and the previously reported one. This is the earliest reported fetus of initial symptoms to date since 11GW and 14GW (Table 3). Given the high expression of the CLTC in various tissues, including the brain, heart, and kidney, neurodevelopmental abnormalities and congenital heart disease were detected in two fetuses. However, the clinical presentations of these conditions varied among the individuals. Previous reports also raised high-echogenic kidneys of the *CLTC* variant (c.3662_3664del) and agenesis of the left kidney and right vesicoureteral reflux (c.2430 + 1G > T). The effect of the *CLTC* variants on renal function deserves our attention. The atrioventricular septal defect appeared in the previous fetus, however, we first observed VSD in the early 11 GW of the present fetus, which was less serious than the previous one. Bilateral choroid plexus cysts of the brain are a new phenotype of the *CLTC* variant.

**Table 3:** Clinical manifestation of the fetuses

Clinical information	Previous fetus	Present fetus
Inheritance	De novo	De novo
Nucleotide change	NM_001288653.1 c.3662_3664del	NM_004859.3: c.3249 + 1_3249 + 168ins
Amino acid change	p.(Leu1221del)	p.Val1084LeufsTer7
	One of the dichorionic diamniotic twins	singleton
**Ultrasound Manifestation**		
11 weeks of gestation		ventricular septal defect 0.02 cm
16 weeks of gestation		bilateral choroid plexus cysts of the brain hyperechogenic kidneys VSD
18 weeks of gestation	fetal growth restriction	
26 weeks of gestation	atrioventricular septal defect; right lateral ventriculomegaly multiple cystic lesions along the left lateral ventricle of the brain	

Our findings contribute to expanding the mutational spectrum of *CLTC* and advance the potential diagnostic windows, offering insights into early disease detection. Furthermore, our study highlights the need for additional cases to elucidate the relationship between genotype and phenotype among *CLTC* variants. The early diagnosis of rare diseases remains challenging, and our research emphasizes the importance of utilizing early and accurate ultrasound scanning and genetic testing to enable early molecular diagnosis. This approach can facilitate early intervention and improve outcomes from birth onwards.

### Electronic supplementary material

Below is the link to the electronic supplementary material.

Supplementary Material 1

## Data Availability

The novel variant c.3249 + 1G > C (NM_004859.3) revealed during the study were submitted to ClinVar database (https://www.ncbi.nlm.nih.gov/clinvar/) under Accession Number SCV004046889.
