# Supplementary material for: A novel de novo CLTC variant altering RNA splicing causes fetal developmental abnormalities
Source: BMC Med Genomics. 2023 Dec 18;16:331. doi: 10.1186/s12920-023-01778-3 (PMC10729518; doi:10.1186/s12920-023-01778-3)

**Supplementary Material**

**sFig.1** The original blot of the Fig 4C. **A** Western blot for antibody of EGFP; **B** Western blot for antibody of GAPDH.


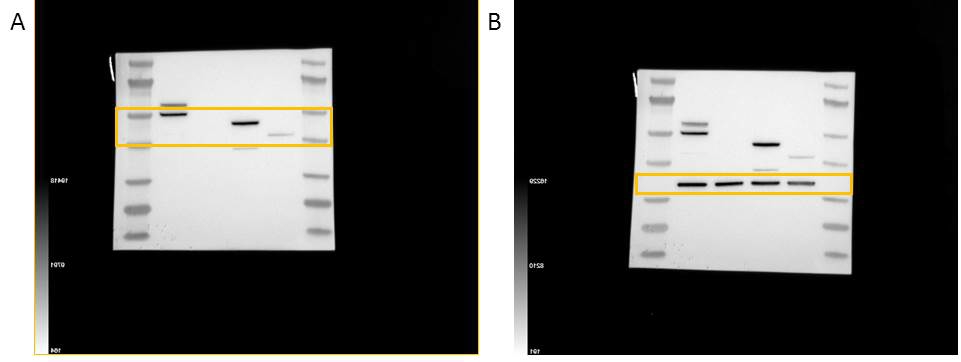

Supplement: Supplementary file 1 — Supplementary Material 1 [file 12920_2023_1778_MOESM1_ESM.docx]
